# Novel *KCNQ2* Variants Related to a Variable Phenotypic Spectrum Ranging from Epilepsy with Auditory Features to Severe Developmental and Epileptic Encephalopathies

**DOI:** 10.3390/ijms26010295

**Published:** 2024-12-31

**Authors:** Mariagrazia Talarico, Radha Procopio, Monica Gagliardi, Maria Chiara Sarubbi, Francesco Fortunato, Ilaria Sammarra, Gaetan Lesca, Donatella Malanga, Grazia Annesi, Antonio Gambardella

**Affiliations:** 1 Institute of Neurology, Department of Medical and Surgical Sciences, University Magna Graecia, 88100 Catanzaro, Italy; m.talarico@unicz.it (M.T.); ilaria.sammarra@unicz.it (I.S.); a.gambardella@unicz.it (A.G.); 2 Neuroscience Research Centre, Medical and Surgical Sciences, 88100 Catanzaro, Italy; radha.procopio@unicz.it (R.P.); monica.gagliardi@unicz.it (M.G.); 3 Laboratory of Molecular Oncology, Department of Experimental and Clinical Medicine, University Magna Graecia, 88100 Catanzaro, Italy; mariachiarasarubbi93@gmail.com (M.C.S.); malanga@unicz.it (D.M.); 4 Genetics Department, Hospices Civils de Lyon, 69000 Lyon, France; gaetan.lesca@chu-lyon.fr; 5 Neuromyogene Institute, Pathology and Genetics of Neuron and Muscle, CNRS UMR 5261 INSERM U1315, University of Lyon—Université Claude Bernard Lyon 1, 69100 Lyon, France; 6 Interdepartmental Center of Services (CIS), University Magna Graecia, 88100 Catanzaro, Italy; 7 Biomedical Research and Innovation, National Research Council, 87050 Mangone, Italy; grazia.annesi@cnr.it

**Keywords:** *KCNQ2*, epilepsy, epilepsy with auditory features, neonatal epilepsy, developmental epileptic encephalopathy, self-limited familial neonatal–infantile epilepsy

## Abstract

Pathogenic *KCNQ2* variants are associated with neonatal epilepsies, ranging from self-limited neonatal epilepsy to *KCNQ2*–developmental and epileptic encephalopathy (DEE). In this study, next-generation sequencing was performed, applying a panel of 142 epilepsy genes on three unrelated individuals and affected family members, showing a wide variability in the epileptic spectrum. The genetic analysis revealed two likely pathogenic missense variants (c.1378G>A and c.2251T>G) and the already-reported pathogenic splice site (c.1631+1G>A) in *KCNQ2* (HGNC:6296). The phenotypes observed in the affected members of family 1, which shared the c.2251T>G variant, were epilepsy with auditory features (EAFs), focal epilepsy, and generalized epilepsy, and none of them suffered from neonatal seizures. The gene panel contained further genes related to EAFs (*LGI1*, *RELN*, *SCN1A*, and *DEPDC5*), which were tested with negative results. The phenotypes observed in family 2 members, sharing the splice site variant, were neonatal seizures and focal epilepsy in childhood. The last unrelated proband, harboring the de novo missense c.1378G>A, presented a clinical phenotype consistent with DEE. In conclusion, we identified two unreported *KCNQ2* variants, and report a proband with EAFs and individuals without typical *KCNQ2* neonatal seizures. Our study underscores the extreme variability in the phenotypic spectrum of *KCNQ2*-related epilepsies and unveils the prospect of its inclusion in screening panels for EAFs.

## 1. Introduction

*KCNQ2* (HGNC:6296) is located on chromosome 20q13.33 and encodes the voltage-gated potassium channel subunit Kv7.2. It is highly expressed in the hippocampus and cortex, in particular at the axonal initial segment [1]. The protein presents the N-terminus and a long C-terminus, both in the cytoplasm, and the transmembrane domain constitutes six segments (S1 to S6). The C-terminus contains four α-helices and intermediate unstructured regions, which are essential for interacting with several proteins for the physiological functioning of the channel [2,3].

Pathogenic variants in *KCNQ2* are notoriously associated with neonatal epilepsies, which can range from a milder phenotype of self-limited neonatal epilepsy (SeLNE) or self-limited familial neonatal–infantile epilepsy (SeLFNIE) to the severe phenotype of *KCNQ2*–developmental epileptic encephalopathy (DEE) [4]. In our recent published Italian survey, *KCNQ2* was reported as the second most prevalent gene for monogenic DEEs [5]. The pathogenic variants in SeLNE or, less frequently, in SeLFNIE, are commonly truncating variants, inherited in an autosomal-dominant way, leading to haploinsufficiency. Conversely, those associated with *KCNQ2*-DEE are usually missense variants arising de novo, leading to a dominant negative effect on the final heterotetramer [2,4,6,7].

Familial neonatal early-onset seizures have been considered as a clinical clue for a *KCNQ2*-related etiology; however, few cases with later childhood epilepsy and no neonatal seizures, as well as intellectual disability, language impairment, and sometimes autistic features, have been reported. These cases have been associated with some missense variants [8,9,10,11] and just one frameshift [12]. All of these few variants have caused a gain-of-function effect, with a poorer prognosis [7].

Thus, the absence or presence of neonatal seizures could represent one of the main discriminants between *KCNQ2* loss- or gain-of-function variants, with a deep impact on precision medicine’s perspectives. The phenotypic spectrum of *KCNQ2*-related epilepsies is now expanding [7,8,9,10,11,12,13].

Epilepsy with auditory features (EAFs) is a focal epileptic syndrome characterized by seizures accompanied by auditory symptoms or receptive aphasia, indicating the possible involvement of the lateral temporal lobe. Genetic involvement in the etiology of EAFs has always been based on the predominance of the *LGI1* gene. Although *LGI1* remains the main causative gene, many other genes have been related to EAF syndrome, including *RELN*, *DEPDC5*, and *SCN1A* [14].

The advent of high-throughput next-generation sequencing (NGS) has facilitated the systematic analysis of epileptic individuals, which has led to the identification of several new variants and risk factors implicated in both epilepsy and encephalopathy [3].

In this study, targeted NGS was performed on three unrelated young epileptic participants, with a wide variability of the phenotypic spectrum, including EAFs, to investigate the genetic background underlying their phenotype.

## 2. Results

### 2.1. Clinical Features

**Figure 1:**
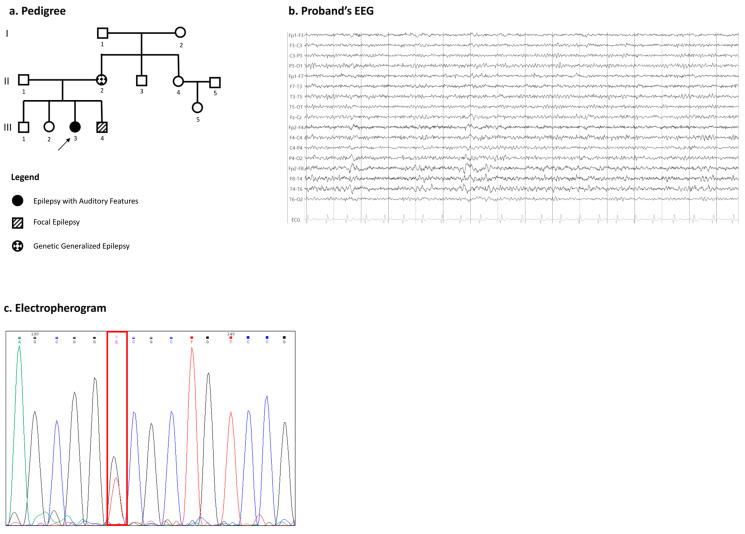
Family 1. (**a**) This section presents the pedigree of family 1. The black arrow highlights the proband. Genetic testing was conducted on individuals II-1, II-2, II-4, III-3, III-4, and III-5, with phenotypic manifestations illustrated in the accompanying legend. (**b**) The proband’s EEG reveals interictal epileptiform discharges originating from the right temporal region (sensitivity: μV/mm; paper speed: 30 mm/s). (**c**) Electropherograms of the proband’s sequence are shown, with the heterozygous nucleotide.

In detail, three members of family 1 were available for clinical evaluation (Figure 1a).

The proband is a 24-year-old right-handed female with a late childhood history of focal epilepsy. She experienced focal auditory-aware seizures ranging from simple elementary sounds (like noises, ringing, and humming) to complex auditory hallucinations. Sometimes these focal seizures impaired her awareness. She did not have febrile seizures, receptive aphasia, or intellectual disability. Neurological examination was unremarkable, a wakefulness EEG showed focal right temporal epileptiform discharges (Figure 1b), and brain magnetic resonance imaging (MRI) was unremarkable. The proband’s brother, with no history of learning disabilities and a normal psychomotor development, had focal epilepsy starting during childhood. Her mother, with no history of intellectual disability and normal psychomotor development, had a history of childhood absence epilepsy (CAE) syndrome, evolving during adulthood into a generalized epilepsy with sporadic tonic–clonic seizures.

**Figure 2:**
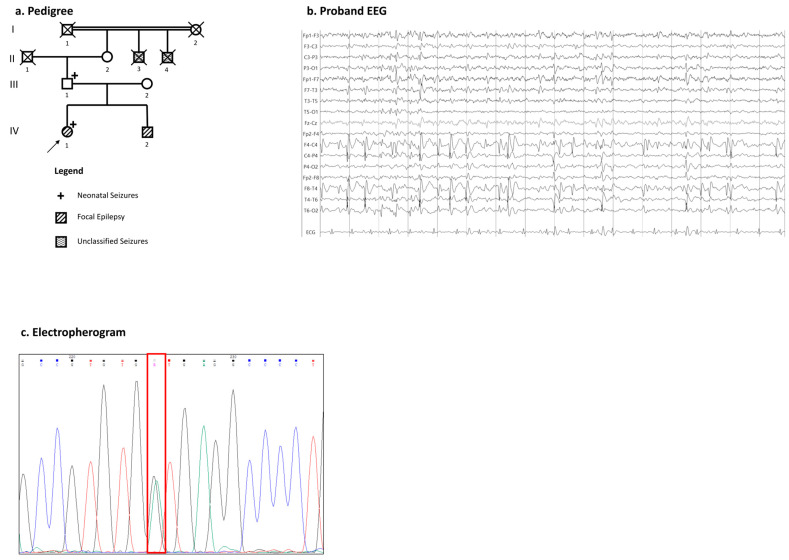
Family 2. (**a**) This section presents the pedigree of family 2, with the proband indicated by a black arrow. Genetic testing was performed on individuals III-1, IV-1, and IV-2; DNA from other family members was unavailable. The legend illustrates the phenotypic manifestations. (**b**) The proband’s EEG displays interictal epileptiform discharges predominantly in the bilateral centro-parietal regions, with a right-side emphasis (sensitivity: μV/mm; paper speed: 30 mm/s). (**c**) Electropherograms of the proband’s sequence highlight a heterozygous nucleotide change at position 1631+1, marked by a red box.

Five members of family 2 were affected with epilepsy (Figure 2a).

The proband is a 21-year-old right-handed female with normal psychomotor development as well as the normal acquisition of the milestones. She experienced tonic–clonic seizures during the first four days of life. At the age of 3 years, she started to manifest, at the same time, the following three different types of afebrile seizures: tonic focal seizures with right head deviation and impaired awareness, eyelid myoclonia, and focal to bilateral tonic–clonic seizures during sleep. The neurological examination was unremarkable, with an intellectual quotient (I.Q.) of 91, and the EEG revealed focal epileptiform discharges located in bilateral centro-parietal regions (Figure 2b). The MRI showed a slight bilateral enlargement of ventricular horns. She started phenobarbital during childhood, with excellent seizure control. Her brother reported a history of focal epilepsy with seizures starting during childhood, which were well controlled by a single monotherapy, and no history of intellectual disability. Her father had only a history of seizures during the first day of life without late-onset epilepsy. The two brothers of the paternal grandmother, deceased and not available for clinical evaluation, also had a history of unclassified epileptic seizures.

**Figure 3:**
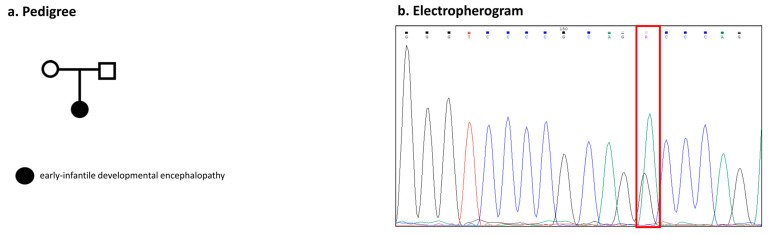
Proband 3. (**a**) The pedigree depicts proband 3 alongside their two unrelated, healthy parents. Genetic testing was conducted on all individuals. (**b**) Electropherograms of the proband’s sequence highlighting a heterozygous nucleotide change at position 1378, marked by a red box.

The other unrelated proband, with an unremarkable familiar history of epilepsy, presented an early-infantile developmental encephalopathy with focal and generalized seizures associated with intellectual disability (Figure 3a).

### 2.2. Molecular Genetic Analysis

This study led to the identification of three variants in KCNQ2 (NM_172107.3). Neither pathogenic nor likely pathogenic variants were detected in other genes analyzed through the panel (Supplementary Materials).

**Figure 4:**
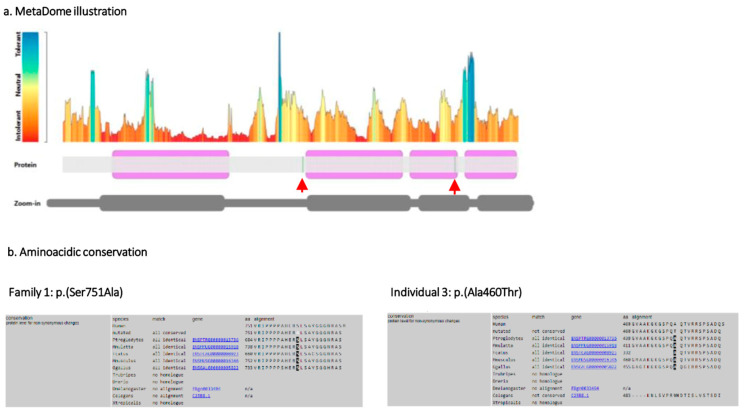
Protein. (**a**) The MetaDome visualization illustrates the protein’s tolerance landscape for missense variants, with the identified variants indicated by red arrows. These variants are in located in regions that are intolerant to missense changes. (**b**) Both figures depict amino acid conservation across species for the two identified missense variants.

In family 1, a missense variant, c.2251T>G, p.(Ser751Ala), was identified in a heterozygous state in exon 17 in the proband (Figure 1c). This variant has never been reported in previous studies. From the same family, the following two affected individuals were analyzed and presented the same heterozygous variant: the mother and the younger brother. Other unaffected relatives resulted to be wild type, including the father, the maternal aunt, and the female cousin. The aminoacidic change occurred in the C-terminus unstructured region and it is predicted to be intolerant to changes (Figure 4a).

The applied predictive tools, summarized in Table 1, resulted as follows: disease causing for MutationTaster; benign for Polyphen2; Revel score, 0.502; CADD-phred score, 21.70; intolerant for MetaDome, with a score of 0.44. The variant has a very low MAF of 0.000003654 on the GnomAD v4.1.0 database and it is absent from the Rikee Project variant database.

According to predictive tools, aminoacidic conservation (Figure 4b), and the ACMG-AMP criteria, we classified this variant as likely pathogenic.

For family 2, the following three members gave consent for DNA testing: the proband, the brother, and the father. In the proband of family 2 was identified the variant c.1631+1G>A in a heterozygous state (Figure 2c), occurring in the first nucleotide of intron 14 and absent from the GnomAD v4.1.0 database. Segregation analysis led to the identification of the variant, also in a heterozygous state, in both her father and brother. The c.1631+1G>A is predicted to highly impact on the donor loss splice site. It negatively affects the splicing in a gene in which loss-of-function is an already known pathogenic mechanism. Predictive tools were applied as described in the methods section and, for this specific variant, it was possible to apply the SpliceVault tool, which predicted a likely exon skipping. According to the ACMG-AMP guidelines, we classified this variant as pathogenic. All of the applied criteria and in silico predictions have been summarized in Table 1.

Moreover, two previous studies reported this variant as pathogenic [15,16], and it has two submissions on ClinVar (National Center for Biotechnology Information; ClinVar, [VCV000369801.4], https://www.ncbi.nlm.nih.gov/clinvar/variation/VCV000369801.4) (accessed on 3 December 2024).

In individual 3 was identified a de novo novel heterozygous missense variant in exon 13, c.1378G>A, p.(Ala460Thr), which was not detected in both of the unaffected parents (Figure 3a,b). This variant is located in an unstructured domain and, according to the MetaDome score, the protein is “slightly intolerant” to the aminoacidic change (Figure 4a). The applied predictive tools results are as follows: disease-causing for MutationTaster; probably damaging for Polyphen2; Revel score, 0.550; CADD-phred score, 21.00; slightly intolerant for MetaDome, with a score of 0.55. The variant has a very low MAF of 0.00000412 on the GnomAD v4.1.0 database and it is absent from the Rikee Project variant database. According to the aminoacidic conservation (Figure 4b), the predictive tools, and following the ACMG-AMP criteria, we classified this variant as likely pathogenic, which was also reported in the two submissions on ClinVar (National Center for Biotechnology Information; ClinVar, [VCV000937790.7], https://www.ncbi.nlm.nih.gov/clinvar/variation/VCV000937790.7) (accessed on 3 December 2024).

A cohort of 100 healthy controls was screened for the candidate variants, which have not been detected in the healthy population.

## 3. Discussion

In the present study, we reported the clinical–genetic features of three unrelated probands, with two novel variants and one already reported *KCNQ2* variant, and a highly variable phenotypic spectrum of epilepsy.

### 3.1. Genetic Findings

In detail, we identified two unreported missense variants, c.2251T>G, p.(Ser751Ala) and c.1378G>A, p.(Ala460Thr), on two unrelated probands. The two variants are in the C-terminus domain. The already reported splice site variant occurs in the distal calmodulin-binding helix. The *KCNQ2* C-terminus domain is a hot-spot region, particularly enriched for pathogenic variants; thus, we have a biological plausibility to claim a deleterious effect of the identified variants. Moreover, we classified all of the variants as “likely pathogenic” or “pathogenic” according to the ACMG criteria.

### 3.2. Phenotypic Variability in Family 1

Our study underscores the extreme variability in the phenotypic spectrum of *KCNQ2*-related epilepsies, ranging from self-limited neonatal seizures to severe developmental encephalopathies. The missense variant c.2251T>G, p.(Ser751Ala) identified in family 1 segregates through the affected individuals and not through the unaffected individuals. Each affected member exhibits a distinct epilepsy phenotype without any neonatal seizure reported: the proband’s brother has a focal epilepsy, the mother had a childhood absence epilepsy, evolving into a genetic generalized epilepsy, while the proband, interestingly, fulfils the clinical criteria for epilepsy with auditory seizures (EAFs) [17]. Although focal epilepsy has been widely reported to be associated with *KCNQ2* variants, to our knowledge, a detailed phenotype with strict auditory features has never been reported. We propose that the variant c.2251T>G, p.(Ser751Ala) may be potentially associated with the proband’s phenotype in family 1, as other possible causes of EAFs, including structural lesions on brain MRI and variants in commonly implicated genes such as *LGI1*, *RELN*, *SCN1A*, and *DEPDC5*, were excluded through screening with negative findings [18]. Other affected family members carry the same variant without presenting the EAF phenotype. Our epilepsy gene panel approach is not without limitations. Thus, we cannot rule out that other genetic factors beyond the *KCNQ2* variant, such as the enrichment of additional rare genetic variants, small deletions, or other structural changes, as well as the influence of a common genomic background, may contribute to determining the EAF phenotype. Nevertheless, the probable involvement of this new *KCNQ2* variant in EAFs cannot be excluded given the results of the molecular analysis. Further studies are needed to verify and clarify the role of this gene in the EAF phenotype. This would not be the first case of missense variants in *KCNQ2* not associated with neonatal seizures; indeed, others have already been reported [8,9,10,11,12]. Considering the cases reported in the literature, we could speculate that our variant has a gain-of-function effect, a result to be functionally confirmed. Moreover, the mother’s proband with the c.2251T>G, p.(Ser751Ala) variant, associated with a classic CAE evolving genetic generalized epilepsy, adds several layers to our understanding. We recognize the polygenic architecture of GGE [19]; however, the description of rare monogenic cases of GGE, as well as larger GWAS studies on “sporadic” cases, are of paramount importance to target potential therapeutic advancements.

### 3.3. Phenotypic Variability in Family 2 and Individual 3

Similarly, in family 2, our investigation led to the identification of a splice site variant, c.1631+1G>A, that segregates through three affected individuals. The proband experienced neonatal seizures during the first four days of life, followed by both focal and generalized seizures during childhood. The proband’s father had only neonatal seizures without a late-onset epilepsy, while the proband’s brother had focal childhood epilepsy without neonatal seizures. In addition, the proband of family 2 was treated with phenobarbital, which is one of the most effective anti-seizure medications in *KCNQ2*-related epilepsy, as well as sodium channel blockers [20]. This variant has been reported in two previous studies [15,16]. Bronwyn et al. reported a family in which the variant segregates through affected individuals, all manifesting heterogeneous phenotypes, including neonatal seizures, febrile seizures, and focal seizures. The variant was documented only in one family with neonatal epilepsy, which was reported by Singh et al. Moreover, splice site variants are primarily associated with a phenotype of SeLNE (or SeLFNIE) [21]. Our data further confirm and reinforce the pathogenicity of this already-reported splice site variant and genotype–phenotype correlations.

The unreported missense variant identified in individual 3 arose de novo, and it is concordant with the most severe phenotypic manifestations. The aminoacidic change falls into a well-conserved domain, and it likely results in a severe functional defect of the channel.

### 3.4. Contribution of This Study

Our description is noteworthy for several reasons. First, we have reported a family whose proband has EAFs, which has never been associated with *KCNQ2* variants. The probable involvement of *KCNQ2* in the EAF phenotype unveils the prospect of its inclusion in gene screening panels. Of course, further investigations are needed to better understand the role of *KCNQ2* in this epileptic syndrome. Secondly, neonatal seizures, which are often considered as a clinical clue for a *KCNQ2*-related etiology, may not always be present, as in the case of our family 1. Thus, we advocate against the “a priori” exclusion of this gene in epileptic people without neonatal–infantile seizures, since it may significantly impact on treatment decisions. Thirdly, our article encourages the importance of performing genetic testing even in adult individuals, a crucial aspect from precision medicine’s perspective. Our study further provides insight into the extreme phenotypic variability observed in *KCNQ2*-related epilepsies, which range from severe, devastating DEEs, as with our individual 3, to milder epilepsies, such as the individuals in families 1 and 2. Notably, we have documented well-defined examples of phenotypic variability both across and within the families. Further studies are needed to evaluate the effects of these variants on the *KCNQ2* channel, as well as to elucidate the role of *KCNQ2* on EAF syndrome.

## 4. Materials and Methods

### 4.1. Participants

Participants were enrolled from our epilepsy clinic of Magna Graecia University of Catanzaro, Italy. The study was approved by the local institutional Ethics Committee (Territorial Ethics Committee of the Calabria Region) and adheres to the principles set out in the Declaration of Helsinki. Informed consent was signed by each participant and/or their legal guardians. Clinical data were collected and de-identified from all probands; then, pedigrees were constructed (Figure 1a, Figure 2a, and Figure 3a). All of the individuals were Caucasian, originating from the south of Italy. Family 1 was composed by the following six members: the female proband, her brother, the two non-consanguineous parents, and her mother’s sister with her son (Figure 1a). Family 2 was composed by the following four members: the female proband, her little brother, and her two non-consanguineous parents (Figure 2a). The other unrelated proband was recruited from our epilepsy cohort (Figure 3a). All of the affected members performed a detailed clinical evaluation with epileptic anamnesis, neurological examination, as well as a standard electroencephalogram (EEG).

### 4.2. Targeted Next-Generation Sequencing 

Peripheral blood was collected and then Genomic DNA was extracted by the Salting Out method. The DNA concentration was evaluated by using the Qubit^®^ 2.0 Fluorometer^®^ (Qubit dsDNA HS Assay Kit, Thermo Fisher Scientific Inc., Waltham, MA, USA).

The NGS analysis was performed on the NextSeq500 (Illumina Inc., San Diego, CA, USA) sequencer, with the settings previously described [22], by applying a gene panel covering the whole coding regions of 142 epileptic genes, including *KCNQ2* (Supplementary Materials).

### 4.3. Bioinformatics Analysis

The identified variants were firstly filtered with specific parameters (total depth > 9; alternative allele depth > 4; no strand bias; mosaicism >10%; GnomAD v4.1.0 database frequency <1%), and then filtered through the databases (dbSNP141; 1000 Genomes Projects datasets; 5000Exome database). Lastly, the variants were annotated by the following functional annotation algorithms: SIFT (http://sift.jcvi.org, accessed on 3 December 2024) and Polyphen2 (http://genetics.bwh.harvard.edu/pph2/, accessed on 3 December 2024).

### 4.4. Sanger Sequencing

To validate the candidate variants, to perform the segregation analysis, and to screen the healthy population, the Sanger sequencing approach was used. The sequencing was performed on the ABI 3500 Genetic Analyzer (Life Technologies, Carlsbad, CA, USA).

### 4.5. Predictive Tools

All of the validated variants were first analyzed through predictive in silico tools, the MutationTaster (https://www.mutationtaster.org/ accessed on 3 December 2024) and Polyphen2 (http://genetics.bwh.harvard.edu/pph2/ accessed on 3 December 2024), to predict the effect on the sequence conservation and protein function. The Revel score [23], CADD-phred score [24], and MetaDome score [25] (https://stuart.radboudumc.nl/metadome/ accessed on 3 December 2024) were employed as predictive tools to assess the probability of the pathogenicity of the two identified missense variants. Additionally, the SpliceVault tool [26] (https://kidsneuro.shinyapps.io/splicevault/ accessed on 3 December 2024), designed to predict the insertion or deletion of splice sites resulting from genetic variants, was utilized to analyze the splice site variant. The GnomAD v4.1.0 database (https://gnomad.broadinstitute.org/ accessed on 3 December 2024) and Rikee Project variant database (https://www.rikee.org/kcnq2-variant-database accessed on 3 December 2024) were investigated. Lastly, we classified the variants according to the ACMG-AMP criteria [27]. All of the predictive analyses and their significance have been reported in Table 1.

## Figures and Tables

**Table 1:** Significance of the scores: Revel: a score from 0 to 1. A score >0.5 is considered pathogenic. CADD-phred: a score >20 is considered pathogenic. MetaDome: a score from 0 to 1. A score between 0 and 0.5 in considered “intolerant”; between 0.5 and 0.7 is considered “slightly intolerant”; >0.7 is considered “neutral”. NA: not available.

Pt n.	Age at Genetic Testing	Variant Type	cDNA Coordinates (NM_172107.3)	Protein Consequences	db SNP	Inheritance	GnomAD v4.1.0	MutationTaster	Polyphen2	Revel	CADD-Phred	MetaDome	ACMG class	ACMG Criteria	Clinical Phenotype
1	18 y	Missense	c.2251T>G	p.(Ser751Ala)	-	Mat.	**0.000003654**	Disease-causing	Benign	**0.502**	**21.70**	**0.44**	**LP**	PM2+PM5+PP1+PP2	**Epilepsy with auditory features. No neonatal seizures.**
2	20 y	Splice site	c.1631+1G>A	p.(?)	rs1057516121	Pat.	**0**	-	-	**-**	**34.00**	**-**	**P**	PVS1+PM2+PP1+PP3+PP5	**Focal epilepsy. Neonatal seizures.**
3	NA	Missense	c.1378G>A	p.(Ala460Thr)	rs1284566908	de novo	**0.00000412**	Disease-causing	Probably damaging	**0.550**	**21.00**	**0.55**	**LP**	PS2+PM2+PP2	**DEE. Neonatal seizures.**

## Data Availability

All of the data generated or analyzed in this study are included in this published article. We submitted the identified variants on the ClinVar database with the following accession numbers: SCV005061841; SCV005061842; SCV005061845.

## Supplementary Materials

The following supporting information can be downloaded at: https://www.mdpi.com/article/10.3390/ijms26010295/s1.
