# Supplementary material for: Novel KCNQ2 Variants Related to a Variable Phenotypic Spectrum Ranging from Epilepsy with Auditory Features to Severe Developmental and Epileptic Encephalopathies
Source: Int J Mol Sci. 2024 Dec 31;26(1):295. doi: 10.3390/ijms26010295 (PMC11719710; doi:10.3390/ijms26010295)
Supplement: Supplementary file 1 [file ijms-26-00295-s001.zip › ijms-3384123-supplementary.pdf]

Article title

Novel KCNQ2 variants related to a variable phenotypic spectrum ranging from epilepsy with auditory features to severe developmental and epileptic encephalopathies

Author list

Mariagrazia Talarico, Radha Procopio, Monica Gagliardi, Maria Chiara Sarubbi, Francesco Fortunato*, Ilaria Sammarra, Gaetan Lesca, Donatella Malanga, Grazia Annesi, Antonio Gambardella

*= corresponding author

Gene panel list

1. *ADSL*
2. *ALDH4A1*
3. *ALDH7A1*
4. *ALG11*
5. *ALG13*
6. *AP3B2*
7. *ARHGEF9*
8. *ARX*
9. *ATIC*
10. *ATP1A2*
11. *ATP1A3*
12. *ATP6V1A*
13. *BRAT1*
14. *BTD*
15. *CACNA1A*
16. *CACNA1E*
17. *CAD*
18. *CASK*
19. *CDKL5*
20. *CHD2*
21. *CHRNA2*
22. *CHRNA4*
23. *CHRNB2*
24. *CLCN4*
25. *CNKSR2*
26. *CPLX1*
27. *CSTB*
28. *CUX2*
29. *CYFIP2*
30. *DEPDC5*
31. *DNM1*
32. *DYRK1A*
33. *EEF1A2*
34. *EPM2A*
35. *FARS2*
36. *FGF12*
37. *FOLR1*
38. *FOXG1*
39. *FRRS1L*
40. *GABBR2*
41. *GABRA1*
42. *GABRA2*
43. *GABRB1*
44. *GABRB2*
45. *GABRB3*
46. *GABRG2*
47. *GNAO1*
48. *GOSR2*
49. *GPHN*
50. *GRIA3*
51. *GRIN1*
52. *GRIN2A*
53. *GRIN2B*
54. *GRIN2D*
55. *HCN1*
56. *HDAC4*
57. *HNRNPU*
58. *IQSEC2*
59. *KCNA1*
60. *KCNA2*
61. *KCNB1*
62. *KCNC1*
63. *KCNH1*
64. *KCNJ10*
65. *KCNMA1*
66. *KCNQ2*
67. *KCNQ3*
68. *KCNQ5*
69. *KCNT1*
70. *KCNT2*
71. *KCTD7*
72. *NEXMIF*
73. *KMT2E*
74. *LGI1*
75. *MBD5*
76. *MECP2*
77. *MEF2C*
78. *MTOR*
79. *NEDD4L*
80. *NHLRC1*
81. *NPRL2*
82. *NPRL3*
83. *NR2F1*
84. *NTRK2*
85. *PACS2*
86. *PCDH19*
87. *PIGA*
88. *PIGG*
89. *PIGN*
90. *PIGO*
91. *PIGQ*
92. *PLCB1*
93. *PNKP*
94. *PNPO*
95. *POLG*
96. *PPP3CA*
97. *PRDM8*
98. *PRRT2*
99. *PTPN23*
100. *QARS*
101. *RARS2*
102. *RELN*
103. *RFT1*
104. *RHOBTB2*
105. *RORA*
106. *RORB*
107. *SCARB2*
108. *SCN1A*
109. *SCN1B*
110. *SCN2A*
111. *SCN3A*
112. *SCN8A*
113. *SIK1*
114. *SLC12A5*
115. *SLC19A3*
116. *SLC1A2*
117. *SLC25A22*

118. *SLC2A1*
119. *SLC35A2*
120. *SLC6A1*
121. *SLC9A6*
122. *SNAP25*
123. *SPTAN1*
124. *SRY*
125. *ST3GAL3*
126. *ST3GAL5*
127. *STAMBP*
128. *STX1B*
129. *STXBP1*
130. *SYN1*
131. *SYNGAP1*
132. *SYNJ1*
133. *SZT2*
134. *TBC1D24*
135. *TCF4*
136. *TPP1*
137. *TRIM8*
138. *UBA5*
139. *UBE3A*
140. *WDR45*
141. *WWOX*
142. *YWHAG*
